# Open-source algorithm for automatic choroid segmentation of OCT volume reconstructions

**DOI:** 10.1038/srep42112

**Published:** 2017-02-09

**Authors:** Javier Mazzaferri, Luke Beaton, Gisèle Hounye, Diane N. Sayah, Santiago Costantino

**Affiliations:** 1 Centre de Recherche Hôpital Maisonneuve-Rosemont, Montréal, QC, Canada; 2 Département d’Ophtalmologie, Université de Montréal, Montréal, QC, Canada

## Abstract

The use of optical coherence tomography (OCT) to study ocular diseases associated with choroidal physiology is sharply limited by the lack of available automated segmentation tools. Current research largely relies on hand-traced, single B-Scan segmentations because commercially available programs require high quality images, and the existing implementations are closed, scarce and not freely available. We developed and implemented a robust algorithm for segmenting and quantifying the choroidal layer from 3-dimensional OCT reconstructions. Here, we describe the algorithm, validate and benchmark the results, and provide an open-source implementation under the General Public License for any researcher to use (https://www.mathworks.com/matlabcentral/fileexchange/61275-choroidsegmentation).

The vessels of the choroid are the exclusive source of oxygen and nutrients to the outer retina and its anatomy plays an important role in the symptoms and pathogenesis of several eye conditions^1^. It has recently been observed that the choroid is thinner in patients with Type I diabetes^2^,^3^, geographic atrophy^4^, pathologic myopia^5^, and retinopathy of prematurity^6^,^7^,^8^. Choroidal visualization based on optical coherence tomography (OCT) has also been used to characterize inflammatory disorders in the posterior segment^9^. Furthermore, choroidal thickness measurements have been shown useful to monitor the progression of patients under VEGF treatment^10^,^11^.

These OCT studies require particularly high signal-to-noise ratio (SNR) images of the deepest layers of the ocular fundus to be able to visualize the choroid clearly. Two technologies have recently enabled this: Enhanced Depth Imaging (EDI) OCT^12^, which is based on an optimal positioning of the reference mirror, and Swept Source-OCT^13^, based on a rapidly tunable infrared laser. However, the choroid topography is delineated manually in the vast majority of studies available in the literature^4^,^10^,^14^,^15^,^16^,^17^. This approach is not only tedious and prohibitively time-consuming, but it is also operator-dependent and fundamentally prone to bias. Moreover, any thorough study of the choroidal topology requires a 3-dimensional description that cannot be performed realistically with manual segmentation. Hence, reliable fully automated segmentation tools are critical to produce comprehensive and statistically relevant studies.

Some modern OCT devices provide automatic segmentation software for the choroid. However, the results depend strongly on the quality of the images, and the software lacks sufficient versatility to adapt to abnormal tissue profiles encountered in many ocular pathologies. A limited group of studies have developed automatic or semiautomatic segmentation algorithms. Zhang *et al*. have developed an algorithm based on the segmentation of individual blood vessels^18^, and this method has been used to study the correlation between the choroidal volume and the level of diabetic macular edema^19^. Kajic *et al*. have proposed a two stage statistical model based on texture and shape analysis to segment the choroid^20^,^21^, which has been applied to assess choroidal thinning in patients with Type-I diabetes^2^ and to segment the Haller and Sattler Layers^22^. DOCTRAP is a patented software, which allows segmentation of several retinal layers, and has been used to measure choroidal thickness in patients with AMD^17^,^23^. Finally, Zhang *et al*. have developed a graph-cut method to segment the choroid layer^24^. Unfortunately, none of these tools is freely available in closed or open-source format, and therefore despite readily available imaging technologies for visualizing the choroid, we believe the lack of available tools to analyze and quantify these images is unnecessarily delaying the progress of the field.

We have recently developed a novel algorithm to automatically detect the choroidal boundaries on OCT time series at a single location in the retina^25^. The method is based on graph theory and we have successfully applied it to measure ocular rigidity by quantifying pulsatile choroidal volume fluctuations at the frequency of the heart. Building on this algorithm, we have extended the computational approach to create choroidal thickness maps from OCT volume reconstructions.

The proposed software segments the anterior and posterior interfaces of the choroid from an OCT volume centered at the macula, and automatically builds a 2D thickness map (3D topology). In this article we describe every step of the algorithm in detail, we assess the software performance, we compare against manual and commercial segmentations, and we demonstrate the use of the algorithm for analyzing the choroid layer in 3D using several surface descriptors.

Finally, in an effort to overcome the shortfall in open-source analysis resources and thereby increase the pace of OCT-based research into ocular pathology, we release the full multiplatform Matlab source code under version 3 of the General Public License https://www.mathworks.com/matlabcentral/fileexchange/61275-choroidsegmentation^26^.

## Segmentation algorithm

The program uses as input a set of OCT images of the retina around the macula and detects the position of 3 layers: the retinal-vitreous interface (RVI), the Bruch’s membrane (BM), and the choroid-sclera interface (CSI). The OCT images consist of a series of B-scans that the algorithm segments individually. The segmentation output of all B-scans is analyzed, combined and finally interpolated to build graphic representations as 2D maps, and allow computation of the thickness of the whole retina as well as that of the choroid.

First, we standardize tomography volume data by representing it as a sorted series of adjacent B-scans and a table describing their positions in the volume. To increase the SNR, images are smoothed using information from adjacent B-scans, retinal interfaces are segmented in each B-scan individually, and finally the 2D retinal and choroidal thickness maps are generated.

### Standardizing tomography images

Different OCT devices have different image storage formats, hence the first step of the algorithm is to convert the tomography into a sorted series of contiguous B-scans and extract their location information with respect to the fundus coordinates. Here we provide the implementation for the Heidelberg-Spectralis format. For other devices, the user needs to extract the B-scans from the device and store them in individual image files which are named to facilitate contiguous sorting, and run a small Matlab script (that we provide) to input basic size and location information.

### RVI and BM segmentation

**Figure 1:**
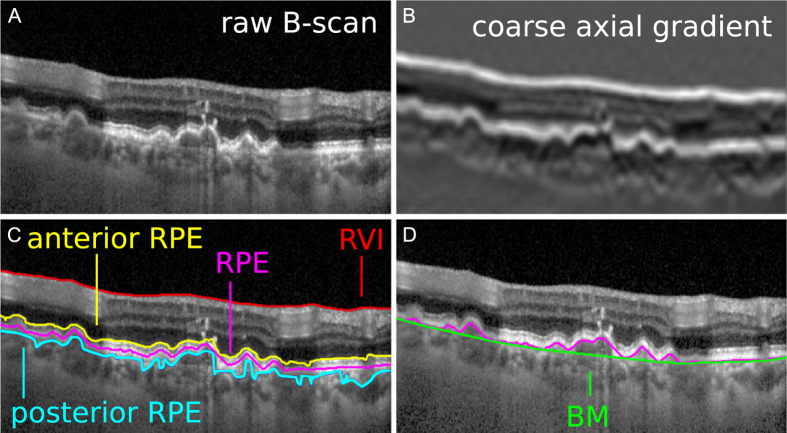
Bruch’s membrane segmentation. (**A**) Raw B-scan. (**B**) Coarse axial gradient illustrating the two most highly contrasted interfaces: RVI and anterior RPE. (**C**) Segmentation of the RVI, first estimation of anterior and posterior RPE interfaces, and the refined segmentation of the RPE center obtained with a graph search algorithm. (**D**) Delineation of the BM as the convex hull of the RPE plus a shift for placing it on the negative intensity slope peak of the RPE (posterior RPE).

The Bruch’s membrane is commonly used to define the anterior limit of the choroid. This membrane, however, is often difficult to distinguish from the RPE in OCT images, particularly for retinas affected by drusen, edema and epithelial dystrophies. Since the RPE is usually easy to segment, we estimate the location of the BM as the convex hull of the RPE. In other words, the convex hull may be interpreted as the shape of a rubber band stretched around the RPE from the posterior side (Fig. 1D).

The strategy to segment the RPE is based on the fact that both the RVI and the RPE display the highest intensity contrast among all interfaces in B-scans (Fig. 1B). A Gaussian low pass filter is first used to smooth out imaging noise and irrelevant small features. We find the interfaces by looking at the two highest local maxima of the intensity gradient in the axial direction, assigning the most anterior peak to the RVI, and the next one to the anterior interface of the RPE. We find a first estimation of the posterior interface of the RPE at the first negative local minima of the intensity gradient below the anterior interface. Outliers are removed from each trace by fitting a 5^th^ degree polynomial and discarding the points that differ from the fit by more than five times the median deviation. The discarded values are replaced using linear interpolation. Next, the center of the RPE in the anterior-posterior direction is localized more precisely and robustly with a graph search algorithm using all pixels between the anterior and posterior interfaces as nodes, and their corresponding pixel intensities as weights (Fig. 1C). Finally, the posterior interface of the RPE is more precisely found at a fixed distance behind the RPE center, where we measure the shift distance by tracing a graph near the RPE but using the absolute value of the intensity slope as weight.

Defining the BM as the convex hull of the RPE posterior interface permits an accurate delineation even when these layers get partially detached, as it is the case of several pathologies.

### Smoothing

**Figure 2:**
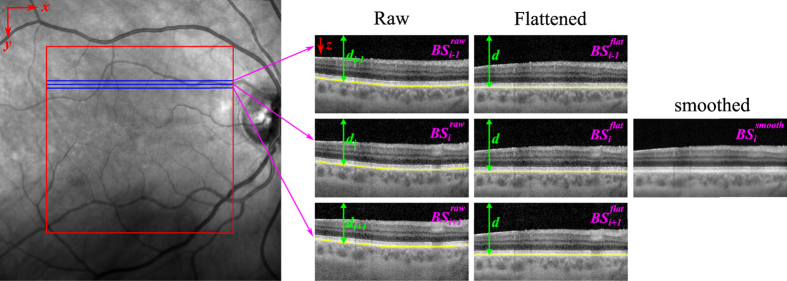
Smoothing scheme. Each B-scan (BS_*i*_) is smoothed in the direction perpendicular to the B-scans (*y*), using a Gaussian weighted average of neighboring B-scans (BS_i+1_, BS_i−1_). Images are registered using the BM as reference. First, the BM is segmented in each raw image (Raw), and then the A-scans are shifted independently to render the BM flat and at the same depth *d* (Flattened). After registration, the smoothed B-scan (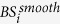) is computed as described in eq. 1.

To increase the SNR, we perform a Gaussian smoothing operation in the transverse direction (*y*) throughout the whole volume using neighboring B-scans (Fig. 2, left). This operation requires registering the images because the axial (z) position of the retina fluctuates between B-scans due to eye movements and accommodation (Fig. 2, Raw).

To do this we use the previously segmented BM as the registration reference, but since the shape of this layer varies between B-scans, the smoothing operation would blur its details. Therefore, we shift the individual A-scans that make up each B-scan to render the BM flat (Fig. 2, Flattened) prior to registration. We compute the smoothed B-scans (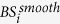) by averaging flattened and registered adjacent images (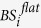) using a Gaussian weight as follows


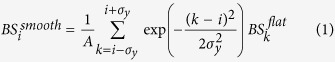


where 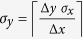, *σ*_*x*_ is the Gaussian averaging parameter in the *x* direction, *Δx* is the pixel size in the *x* direction, *Δy* is the distance between B-scans, the operator 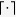 returns the smallest integer greater than or equal to its argument, and 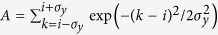. A Gaussian averaging in *x* and *z* directions is performed at a later step.

### CSI segmentation

**Figure 3:**
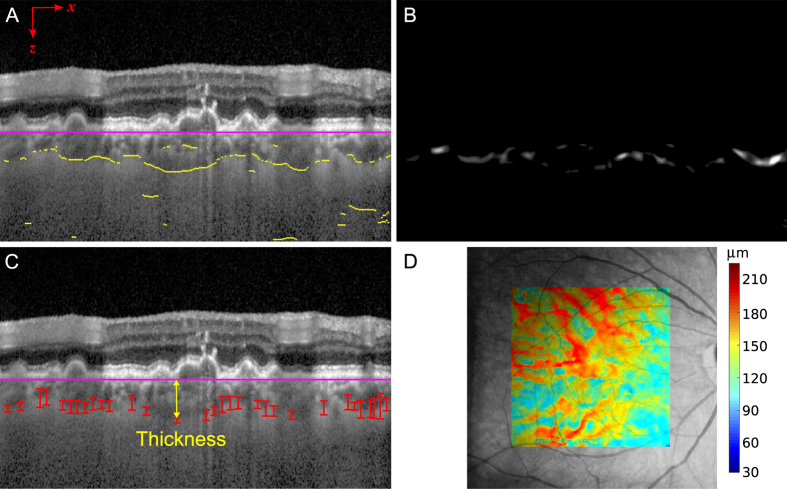
CSI segmentation using a graph-search approach. (**A**) The nodes of the graph are found as inflexion points of intensity in the *z* direction, below the BM. (**B**) Edge probability *P(x,y*), based on the Gabor transform, and used to compute the weight component *w*_*Affin*_. (**C**) Nodes resulting from the graph-search, delineating the CSI. The bars indicate the edge probability of each node and the arrow illustrates the computation of the choroidal thickness. (**D**) Color coded choroidal thickness map obtained by natural neighbor interpolation of thickness on the scattered nodes along all B-Scans, overlaid on the fundus image.

Using these SNR-improved B-scans, and extending on our previous work, the CSI is segmented on each B-scan using an algorithm based on graph searching^25^. First, the image is rescaled to the [0, 255] range, and smoothed in *x* and *z*, using a Gaussian low-pass filter with parameters (*σ*_*x*,_ *σ*_*z*_). The candidate nodes of the graph are defined as the pixels where the derivative of the intensity with respect to *z* is greater than a predefined positive threshold and the absolute value of the second derivative is near zero (i.e. lower than another very small threshold). In other words, the nodes are defined as the inflexion points of the intensity in the z direction, which localize to the posterior walls of blood vessels where intensity transitions from dark to bright (Fig. 3A) as the A-scan passes from vessel to sclera.

For the graph-search we assign weights *w(a,b*) to the connections between nodes *a* and *b*, defined as:


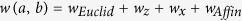


The Euclidean distance between nodes *w*_*Euclid*_ favors connecting nodes close to each other. The jump penalties *w*_*z*_ and *w*_*x*_ are defined as


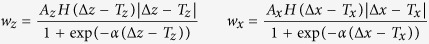


to discourage large leaps in *z* and *x* directions respectively, where *A*_*x*_, *A*_*z*_, *T*_*x*_, *T*_*z*_, and *α* are parameters and *H(x*) is the Heaviside step function. Finally, *w*_*Affin*_ favors bonds going through regions of high edge probability *P(x,z*). This probability is computed using the Gabor transform 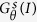, which provides an estimation of the derivative at the angle *θ* and at scale *s*. It is defined as the correlation between a Gabor function 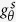 and the image *I*, where


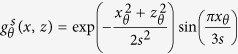


and (*x*_*θ*_, *z*_*θ*_) are coordinates in a system rotated by an angle *θ* with respect to (*x, z*) according to


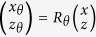


where *R*_*θ*_ is a rotation operator. The edge probability is finally computed pixelwise as


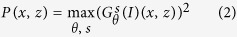


where the maximum is taken from a set of Gabor transforms at angles in a range [−20^o^, 20^o^] around the positive *z* direction, and scales in the range [10 *μ*m, 20 *μ*m]. The probability is normalized to the range [0, 1] (Fig. 3B). The weight *w*_*Affin*_ is computed as 1/*A*, where *A* is the line integral of *P(x,z*) along pixels in a straight line *L* connecting nodes *a* and *b* as


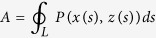


Only nodes spaced less than 5*T*_*z*_ in *z* and 5*T*_*x*_ in *x* were allowed to connect.

For the start and end nodes, we added two virtual nodes before the first and after the last columns respectively. The minimum weight path through the graph is found using the Dijkstra’s algorithm^27^ (Fig. 3C).

Sometimes, however, only a few vessels are clearly visible in the B-Scan, and the number of candidate nodes found is not enough to produce a whole trace across the B-scan. In order to profit from those nodes detected, and when a full-length trace fails, we subdivide the graph into connected fragments and search for the shortest path within each fragment. We use Tarjan’s algorithm for the subdivision^28^, and after running Dijkstra’s algorithm on each sub graph the resulting path segments are analyzed for consistency. This involves identifying regions in the *x* axis where some segments overlap, and discarding unlikely segments to avoid superposition.

To make this choice, we assign weights to each segment based on their edge likelihood, computed as follows:


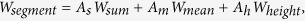


where *W*_*sum*_ is the sum of the edge probability *P* (eq. 2) along all nodes, *W*_*mean*_ is the mean probability, *W*_*height*_ is one over the mean height of the segment in *z*, and *A*_*s*_, *A*_*m*_, and *A*_*h*_ are arbitrary coefficients adding up to one. The CSI is finally computed by joining the nodes from the remaining path segments.

The choroidal thickness is computed at each node as the distance between BM and CSI. After computing the value for all nodes from every B-Scan, we use the 2D natural neighbor interpolation method to estimate the thickness of the choroid on a grid of points within the limits defined by the position of the B-scans on the fundus of the eye (Fig. 3D)^29^.

## Results

### Performance analysis

Despite a robust design, the performance of the method depends on image quality and on the health of the retina. Very low signal-to-noise-ratio images, retinas containing large amounts of drusen, or significant damage to the RPE may hinder successful segmentation. Cases where medical specialists are unable to distinguish retinal layers are rarely traced accurately by the software. For images with low signal to noise ratio, or where choroidal vessels are not well defined, CSI segmentation fails at a higher rate.

**Figure 4:**
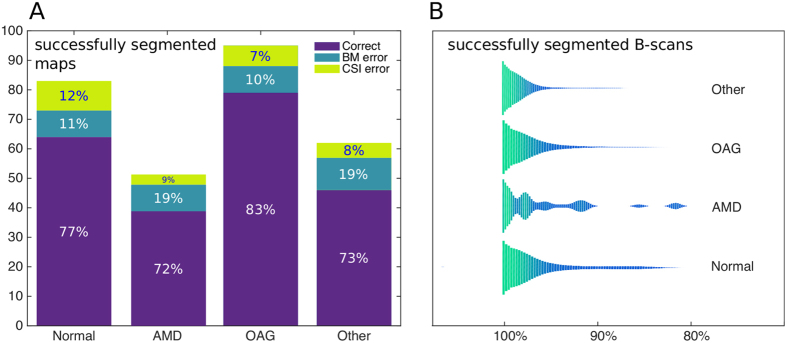
Segmentation performance. (**A**) Number of correctly segmented choroidal thickness maps grouped by eye condition, for a total of 280 patients. (**B**) Violin plots depicting the distribution of the fraction of successfully segmented B-scans per map, for all successfully reconstructed maps (215 cases), described in separate patient cohorts.

For an overall assessment of the segmentation success rate, we applied the method to an image database of 280 patients with various eye conditions from the ophthalmology clinic at the Maisonneuve-Rosemont Hospital, Montreal, Québec, Canada. The study protocol adhered to standards outlined in the Declaration of Helsinki, and all participants signed an informed consent approved by the “Comité d'éthique de la recherche de l’installation de l’Hôpital Maisonneuve-Rosemont du CIUSSS de l’Est-de-l’Île-de-Montréal”. We have examined all maps visually, and guided by outlier regions in the thickness maps we have identified 65 problematic cases. Of these 65 cases, the source of inaccurate map generation resulted from incorrect BM segmentation and CSI segmentation failure in 38 and 27 maps respectively, evenly distributed among patient cohorts (Fig. 4A).

In the 215 successfully reconstructed maps, we additionally studied the fraction of B-scans successfully segmented. All of these reconstructions showed accurate delineation of the BM. As for the CSI, we calculated the number of B-scans in a map where at least one CSI segment containing at least 4 nodes was found, and plotted this distribution in Fig. 4B, grouped by disease. For all patient cohorts, the mean successful fraction is always above 96% with standard deviation below 5%. Notably, there is a larger fraction of B-scan segmentation errors for AMD patients (3.5%) compared to normal subjects (1.5%). This 2% difference between AMD and Normal patients is completely due to errors in BM detection (2.5% for AMD and 0.5% for normals), where the segmentation process is hampered by strongly damaged RPE layers.

### Validation of segmentation

We have also validated the method quantitatively by comparing our results with both manual segmentations and the commercial software included in Heidelberg Spectralis OCTs. Since there is no gold standard for assessing the accuracy of segmentation of the retinal layers, we defined as ground truth the tracings made by experienced eye-specialists^25^.

To produce manual segmentations, we selected 30 B-Scans at random from a list of patients with diverse conditions including wet and early AMD, glaucoma, uveitis and normal (healthy) subjects; these 30 raw B-Scans were presented sequentially and twice over (once for tracing the BM and once for the CSI) to a group of 5 independent evaluators who were asked to delineate the layers manually. The evaluators manually segmented the BM and the CSI directly on the touch screen of a tablet computer (Samsung Galaxy Note 10.1, Model SM-P600), using a stylus pen. The two traces were drawn using pre-set colors and analyzed to extract the manual segmentations.

**Figure 5:**
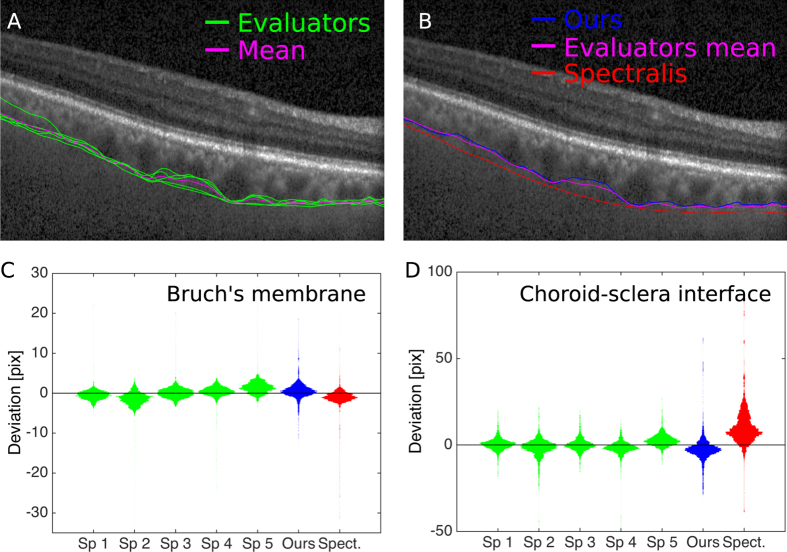
Segmentation comparison. (**A**) Manual CSI segmentations and their mean trace. (**B**) Sample CSI traces of the ground truth (evaluator mean), our method, and Spectralis algorithm. We studied the differences between each segmentation and the ground-truth trace, which we compute as the mean trace of all specialists. For each specialist and the two automated methods, we compute the deviation (from ground truth) at all A-Scans of all tested B-Scans and present the results as violin plots. (**C**) Comparison of Bruch’s membrane segmentation. (**D**) Comparison of choroid-sclera interface segmentation.

In order to compensate for inter-subject variability, we defined the ground truth for a particular layer as the mean trace among the five evaluators (Fig. 5A), and all segmentation algorithms and individual evaluators are compared to this average (Fig. 5B–D). The process starts by localizing the pixels displaying the annotation color (*x*_*c*_, *z*_*c*_). At each column *x*_*c*_ we compute the mean 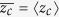 and standard deviation 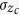 to estimate the position and thickness of the trace in each column. The line thickness is estimated as 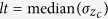 across all columns. After discarding columns where 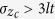, we obtained the trace coordinates (*x*_*s*_, *z*_*s*_) by interpolating the remaining 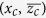 with splines (Fig. 5A).

The traces produced by the five subjects were averaged at each column to render the ground truth trace 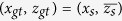 as shown in Fig. 5A. When assessing the segmentation accuracy of both manual and automated methods for a given B-scan, we computed the distance between any particular trace and the corresponding ground truth at each column (A-Scan) for all images, and we analyzed the statistical distribution of these distances. In Fig. 5C and D (for the BM and CSI respectively), we plotted the distribution of deviations from the ground truth for all the evaluators, our method, and the Spectralis commercial software.

The distribution of deviations for the commercial software and our method is largely equivalent to manual evaluators regarding segmentation of the BM, where all distributions medians are smaller than 6 μm. For the CSI, the performance of our method is similar to manual segmentation (all medians are smaller than 11 μm), whereas the commercial software produces larger deviations (more than 30 μm. See Fig. 5D), possibly due to over-smoothing (as appreciated in Fig. 5B).

### Three-dimensional study of the choroid

Three-dimensional information permits the study of the choroid along new, unexplored avenues. Our open algorithm provides a reliable tool for approaching these studies on large patient databases, which are in turn necessary to uncover novel disease biomarkers with statistical significance.

**Figure 6:**
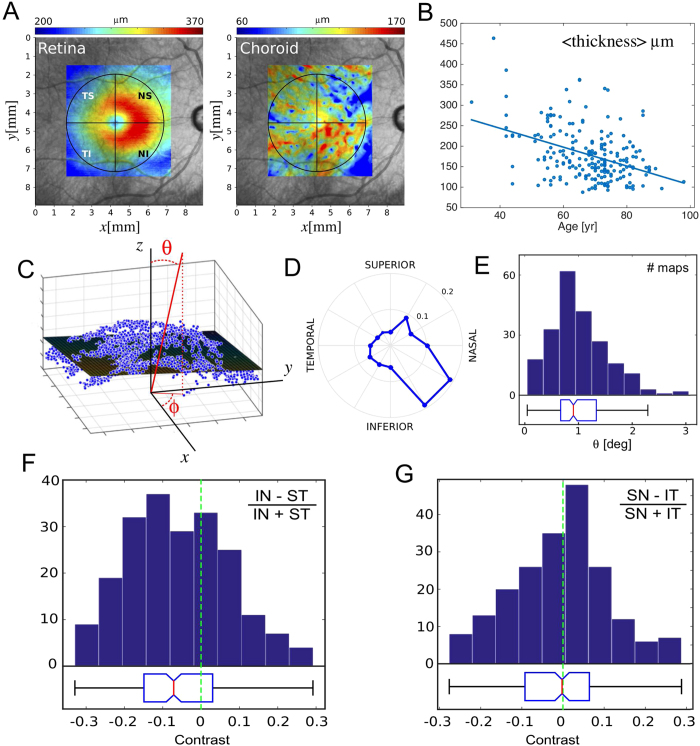
Three-dimensional study of the choroid. (**A**) Retina thickness map, computed as the distance between the RVI and BM, and choroidal thickness map. A circular region is defined to extract data centered at the macula. (**B**) Mean choroidal thickness versus age. (**C**) Scatter plot of choroidal thickness in 2D with fitted first-degree 2D polynomial. The normal to the plane is described by the angles *θ* and *ϕ* in spherical coordinates. (**D**) Polar normalized histogram of the azimuthal angle *ϕ* for the whole set of patients. (**E**) Histogram of the polar angle, with median value near 1 degree. (**F**) and (**G**) Histograms of the thickness contrast between inferior-nasal and superior-temporal, and between superior-nasal and inferior-temporal. These contrasts are consistent with the tilt azimuthal angle of plane fit to the map.

In order to illustrate this, we applied the algorithm to a database of 215 patients (129 women and 86 men), spanning a range of ocular disease (63 normal, 43 AMD, 76 OAG, 28 Uveitis, 14 Other). The average age of the cohort is 68 years old with a standard deviation of 11. The image dataset consists of OCT scans over square regions (7.5 × 7.5 mm^2^) approximately centered at the macula (Fig. 6A), composed of either 50 (101 volumes) or 192 B-scans (114 volumes) spaced accordingly.

We have analyzed the resulting choroidal thickness maps in circular regions centered at the macula (Fig. 6A). By subtracting the RVI from the BM traces in every B-Scan, we have built a map of retinal thickness (Fig. 6A), and we found the coordinates of the local minimum to locate the center of the macula. The largest possible circle centered on this minimum and still bounded by the original scanned region was used as region of interest in every calculation. Overall, the mean diameter of these circles is 5.3 mm, with standard deviation of 0.4 mm, and a minimum value of 3 mm.

Maps consist of a set of scattered points (*x*_*k*_,*y*_*k*_) within the circular region indicating choroidal thickness (*Δz*_*k*_), and a weight (*W*_*k*_) calculated as the edge probability P defined by eq. 2. Using this dataset, we have computed a set of anatomical descriptors.

First we have calculated the mean choroidal thickness using a weighted average as follows


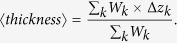


We observe that the average thickness correlates negatively with age (Fig. 6B, R^2^ = 0.17, p = 10^−10^), and decreases approximately 23 μm per decade, similar to what has been previously reported using manual measurements on single-scan OCT images (10 μm/decade^30^, 15.6 μm/decade^31^, and 14 μm/decade^32^). Similarly, when only normal patients are considered, the choroid mean thickness decreases 27 μm per decade (R^2^ = 0.16, p = 10^−3^) suggesting that this measurement is not biased by age related pathologies.

We have also fit a plane (2D first-degree polynomial) to the thickness map, as illustrated in Fig. 6C, and analyzed the distribution of the angles *θ* (polar) and *ϕ* (azimuthal) describing the direction of the vector normal to such plane. These values indicate the tilt and orientation of choroidal thickness. Figure 6D shows a normalized histogram of the azimuthal angle, oriented to the inferior-nasal quadrant, which reflects that the choroid is thinner in this region. Barteselli *et al*. have reported results consistent with this observation using manual segmentation of OCT volumes. They report that the nasal quadrant has the lowest choroidal volume, and the superior quadrant presents the highest^33^. We have also computed the distribution of thickness contrasts between the inferior-nasal and the superior-temporal quadrants (Fig. 6F), along with the contrast between the superior-nasal and the inferior-temporal (Fig. 6G), which confirm the results in Fig. 6D.

## Discussion

The vast majority of studies based on choroidal thickness maps published so far, which will become seminal for the determination of therapeutic decisions, are based on software black boxes. Due to the variety of eye pathology, the diversity of tissue shapes, and the limitations on image quality (particularly in elderly patients), it is rare that a single piece of software performs optimally in all cases. Open-source algorithms, such as the one we present here, provide the necessary flexibility and freedom as well as long term public support, whereas proprietary software packaged exclusively with new hardware hinders progress in a field in which manual measurements of large cohorts is not a feasible segmentation alternative.

The segmentation strategy we present also possesses some compelling characteristics. Although the CSI segmentation is based on graph theory, it does not require a fully connected path across an entire B-scan to be successful. Given the tortuosity and variable reflectivity of blood vessels in the choroid, sometimes only a few of them are visible in the images; it is therefore beneficial to make use of all available information to reconstruct the topography. The approach also benefits from averaging adjacent B-Scans to obtain higher signal-to-noise-ratio images for improved segmentation results.

Additionally, although a 2D scattered interpolation is used to build the visual representation of the maps, quantifications are based solely on detected graph nodes weighted by the edge probability, preventing interpolation artifacts from interfering with results.

The method is also highly robust, overcoming to a large extent the segmentation challenges posed by tissue anomalies such as drusen and RPE discontinuities, and by low quality images. This is reflected in the high number of correctly segmented maps (Fig. 4A), and it is explained by the substantial fraction of successfully segmented B-Scans per volume (Fig. 4B). Validation against manual tracing demonstrates that the segmentation accuracy performs at least as well as specialists, and it outperforms the software provided by the very device used to capture its input images.

The software we provide is coded in Matlab, which is multiplatform and is widely used for quantitative analysis of biomedical studies. The code has been tested on Matlab R2014b for OSX El Capitan 64bits, Matlab R2014a for Linux 64 bits, and Matlab 2013b for Windows 10 64 bits.

The systematic analysis of the role of the deepest layers of the eye in the pathogenesis of disease is becoming the focus of an impressive number of studies. Beyond average choroidal thickness, the analysis of 3-dimensional surfaces opens the door to a panoply of local and global descriptors that may correlate with ocular physiology, and which are presently unexplored. Accurate and flexible tools for delineation of the choroid are essential to find these anatomical and functional biomarkers of the progression and outcome of blinding disorders.

## Additional Information

**How to cite this article:** Mazzaferri, J. *et al*. Open-source algorithm for automatic choroid segmentation of OCT volume reconstructions. *Sci. Rep.* **7**, 42112; doi: 10.1038/srep42112 (2017).

**Publisher's note:** Springer Nature remains neutral with regard to jurisdictional claims in published maps and institutional affiliations.
